# Meta-Analysis of MINIject vs. Two iStents as Standalone Treatment for Glaucoma with 24 Months of Follow-Up †

**DOI:** 10.3390/jcm13247703

**Published:** 2024-12-17

**Authors:** Jeremy C. K. Tan, Ashish Agar, Harsha L. Rao, Katherin Awad, Kaweh Mansouri

**Affiliations:** 1 Faculty of Medicine and Health, University of New South Wales, Kensington, NSW 2032, Australia; jeremy.c.tan@unsw.edu.au (J.C.K.T.);; 2 Department of Ophthalmology, Prince of Wales Hospital, Randwick, NSW 2031, Australia; 3 Narayana Nethralaya, 63, Bannerghatta Road, Hulimavu, Bangalore 560076, India; 4 University Eye Clinic Maastricht, University Medical Center, 6229 HX Maastricht, The Netherlands; 5 iSTAR Medical, 1300 Wavre, Belgium; katherin.awad@istar-medical.com; 6 Swiss Visio Glaucoma Research Center, Montchoisi Clinic, 1006 Lausanne, Switzerland; 7 Glaucoma Department, University of Colorado Denver, Denver, CO 80045, USA

**Keywords:** glaucoma, minimally invasive surgery, intraocular pressure, suprachoroidal space

## Abstract

**Background:** This study compares the long-term intraocular pressure (IOP)-lowering efficacy of standalone MINIject (iSTAR Medical, Belgium) suprachoroidal implantation and two iStent (Glaukos, CA, USA) trabecular bypass implantation using a systematic review and meta-analysis. **Methods:** Systematic review of standalone implantation of MINIject or iStent inject with at least 24 months of follow up. The mean and standard deviation of IOP and the number of IOP-lowering medications at baseline and at 24 months were extracted. Weighted estimates of the outcome variables were calculated using random-effects meta-analysis models. Heterogeneity in the outcome measures among the studies was quantified using I². **Results**: Seven studies (three studies for MINIject and four for iStent) comprising 280 eyes were included. At 24 months, there was a greater reduction in IOP from baseline in the MINIject vs. two iStent cohorts (−9.57 vs. −4.92 mmHg, *p* = 0.03). The change from baseline in mean medication use was −1.00 with MINIject and −0.56 medications with iStent (*p* = 0.26). The mean percentage IOP reduction at 24 months ranged from 36.3−42.2% with MINIject compared to 5.2−40.7% with iStent, with greater variability in mean change from baseline in IOP observed in the iStent group (I^2^ = 96.5% vs. 0%). The most frequent adverse events for MINIject were anterior chamber inflammation, best-corrected visual acuity (BCVA) loss, hyphema, and conjunctival hemorrhage, and for iStent, these were device obstruction, BCVA loss, IOP spike, and cataract progression. **Conclusions:** While both MINIject and iStent inject devices resulted in significant reductions in IOP and IOP medication use, standalone MINIject may provide a greater and more consistent reduction in IOP.

## 1. Introduction

Glaucoma is a group of disorders characterised by the progressive degeneration of retinal ganglion cells and is the leading cause of irreversible blindness in the world [1]. Intraocular pressure (IOP) is the only known modifiable risk factor for the treatment of glaucoma. IOP reduction is usually achieved by topical IOP-lowering medications and laser therapy initially, with surgical procedures reserved for patients with more advanced disease and where IOP is uncontrolled with conservative treatment. Over half of patients placed on topical medications are, however, nonadherent by 3–4 years of treatment [2], which arises from factors such as the cost of therapy, side effects, and forgetfulness [3]. Strategies have been explored to develop long-acting, minimally invasive therapies for glaucoma [4]. Minimally invasive glaucoma surgery (MIGS) refers to a new class of devices which shunt aqueous humour from the anterior chamber into the Schlemm’s canal, suprachoroidal space, or subconjunctival space [5]. The latter results in the formation of a bleb; therefore, there is debate on whether these devices can be classified under the definition of MIGS [6,7]. Besides IOP reduction, these methods can reduce or eliminate the need for topical medications. MIGS devices are considered to be safer than conventional filtration surgery and may, therefore, assume an important role in the treatment paradigm of glaucoma [8]. The clinical and economic effectiveness of MIGS devices, however, remains unclear due to the paucity of large randomised controlled trials and observational studies [9].

The iStent^®^ and iStent^®^ inject devices (Glaukos Corporation, San Clemente, CA, USA) are trabecular micro-bypass stents which are the most commonly performed MIGS procedure in the USA [10]. The first-generation iStent was the first MIGS device to be approved by the US Food and Drug Administration (FDA). It has been widely demonstrated to provide effective lowering of IOP alone or in combination with phacoemulsification [11,12]. The second-generation iStent inject was approved by the US FDA in 2018 and involves the implantation of two trabecular stents [11]. MINIject^®^  (iSTAR Medical SA, Wavre, Belgium) is a new device measuring 5 mm in length with a cross-sectional dimension of 1.1 × 0.6 mm, and is designed for insertion into the supraciliary space via an ab interno procedure [13]. It is made of biocompatible silicone and features a porous design to encourage aqueous flow and reduce the incidence of fibrosis [13]. Early studies have demonstrated effective reduction in IOP and IOP medication use in the first two years [14,15].

While numerous studies have shown the IOP efficacy of MIGS devices combined with phacoemulsification, the magnitude of IOP lowering obtained with MIGS devices alone is less clear. Cataract surgery can itself decrease IOP in patients with elevated IOP [16]; therefore, studies on standalone MIGS are important to help us better understand the effect of these devices alone on IOP. Furthermore, while studies often report the IOP efficacy of MIGS devices at 12 months, few studies report longer-term outcomes. This study aims to examine and compare the long-term IOP-lowering efficacy of a supraciliary drainage device (MINIject) and trabecular bypass device (two iStent) independent of the impact of cataract surgery. We therefore performed a meta-analysis of standalone MINIject studies vs. iStent studies with a follow-up duration of at least 24 months. 

## 2. Materials and Methods

**Figure 1:**
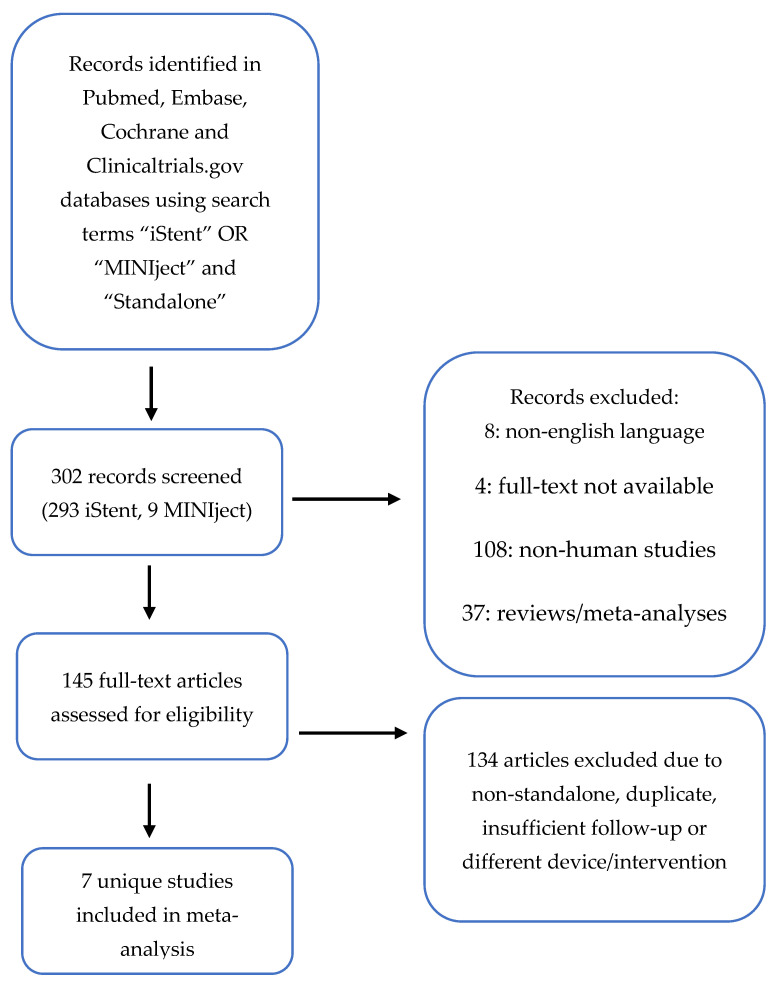
Preferred Reporting Items for Systematic Reviews and Meta-Analyses flow chart showing literature search.

A search of the PubMed, ScienceDirect, Cochrane Library, Embase, and ClinicalTrials.gov databases was conducted in March 2023 for studies of MINIject and iStent. This systematic review was performed according to the guidelines set out in the Preferred Reporting Items for Systematic Reviews and Meta-Analyses statement (Figure 1) The search terms used were “MINIject”, “iStent”, and “standalone”. Inclusion criteria were full-text articles published in English, human studies, and prospective or retrospective studies including patients undergoing standalone implantation of a single MINIject or two iStents, with two years of follow up. The longest duration of follow-up was limited in the supraciliary implant group, with a maximum follow up of 2 years. Because this was a meta-analysis of the efficacy of IOP lowering between both standalone devices, we therefore decided to use this common time point available in both sets of studies. Studies which reported the outcomes of both standalone procedures and procedures combined with cataract surgery were included if the outcomes for standalone procedures were reported separately. Likewise, the device arm of studies randomizing or comparing the device to other technologies was also included. To supplement published data on MINIject, internal data on individual trials from the manufacturer database were requested, and results were included for all studies where 24-month data were available. Exclusion criteria were the following study designs: systematic review, meta-analysis, pooled-analysis, review, and guidelines.

### 2.1. Parameters Analysed and Definitions of Success

The following baseline parameters and outcome measures were extracted from the identified studies: demographics (age, gender, ethnicity, number of patients); baseline ophthalmic characteristics such as lens status (phakic, pseudophakic); and efficacy data. Efficacy data included mean and standard deviation (SD) of IOP at baseline, 6, 12, and 24 months; mean and SD of number of IOP-lowering medications at baseline, 6, 12, and 24 months; percentage IOP reduction at 6, 12, and 24 months; and percentage of patients with IOP ≤ 18 mmHg at 24 months. When SD was not directly reported, it was calculated either from 95% confidence intervals or from standard errors, whichever was available. When all of these were not available in text or tables, they were derived from the graphs present in the manuscript. When longer-term data for studies were not yet published, they were extracted from company press releases, where available. If baseline and 24-month mean and standard deviations for IOP and medication use were not available despite exhausting all possible avenues to find the relevant data, the article was excluded.

### 2.2. Meta-Analysis of MINIject vs. iStent IOP Outcomes

MINIject and iStent studies reporting reductions in IOP and medication outcomes at 24 months were included in the meta-analysis. Weighted estimates of the outcome variables at 24 months were calculated using random-effects meta-analysis models. The results of the meta-analysis are presented using forest plots. Mean treatment differences with *p*-values were reported with the following outcomes: “mean change from baseline in IOP” and “mean change from baseline in IOP-lowering medication use”. Heterogeneity in the outcome measures among the studies was quantified using multiple parameters such as I², τ², H², and the test of homogeneity [17]. The Risk Of Bias In Non-Randomized Studies—of Interventions (ROBINS-I) tool (available at Risk of bias tools - ROBINS-I tool (google.com) Accessed on 1 June 2023) was used to assess the risk of bias in the results of the identified studies.

Appropriate statistical methodology of key clinically meaningful outcome analyses was conducted. This included using random effects methodology and t-test comparisons to provide an overall calculated assessment of relative efficacy. The *p*-values were calculated from the meta-analyses. A *p*-value of ≤0.05 was considered statistically significant. Statistical analyses were performed using the Stata version 17.0 (StataCorp, College Station, TX, USA) statistical software.

## 3. Results

Seven studies (three studies for MINIject [13,14,15] and four for iStent [8,18,19,20,21,22]) comprising 280 eyes satisfied the requirements and reported relevant data and, thus, were included in the meta-analysis. The three MINIject studies were all prospective, single-arm clinical trials, and the iStent studies were a combination of prospective and retrospective studies.

### 3.1. Baseline Patient Characteristics

The key baseline patient characteristics, including age, gender, number of pre-operative medications, pre-operative IOP, and participant sample sizes and diagnostic inclusion criteria for the three MINIject and four iStent studies, are shown in Table 1.

Two of the three MINIject studies were conducted in non-White populations, while one study was conducted in a European population comprising 81% Caucasian and 13% Black individuals. The iStent studies were mainly conducted in White populations. The mean baseline IOP and number of IOP-lowering medications was 23.7 mmHg (range 23.2–24.6) and 2.4 (range 2.0–3.0) in the MINIject studies and 21.1 mmHg (range 19.1–25.3) and 2.7 (range 2.2–3.0) in the iStent cohort, respectively (*p* > 0.05). The meta-analysis results for baseline IOP and medication-use can be found in supplementary Figure 2 and Figure 3. The three MINIject studies included patients with primary open-angle glaucoma or open-angle glaucoma, while two of the four iStent studies also included pseudoexfoliative and other secondary glaucomas [19], as well as ocular hypertension [20].

Table 2 displays the IOP-lowering outcomes reported in the MINIject studies at baseline, 6, 12, and 24 months. At 24 months post-operatively, the mean IOP ranged from 13.6 mmHg to 15.5 mmHg, and the mean percentage IOP reduction ranged from 36.3% to 42.2%. The percentage of participants with IOP values of 18 mmHg or less ranged from 74% to 95%, while the mean number of medications ranged from 1.0 to 1.8 at 24 months.

Table 3 displays the IOP-lowering outcomes reported in the iStent studies at baseline, 6, 12, and 24 months. The mean IOP at 24 months post-operatively ranged from 15.0 mmHg to 18.1 mmHg. The mean percentage IOP reduction ranged from 5.2% to 40.7%. Three studies reported the mean number of medications at 24 months, which ranged from 1.7 to 3.0. Only one of the four studies reported the percentage of participants with IOP values of 18 mmHg or less at 24 months.

### 3.2. Meta-Analyses of Mean IOP Reduction in MINIject vs. iStent up to 24 Months

Outcomes from the identified studies were extracted, analysed, and reported in the forest plots presented below. The mean IOP values at baseline and at 24 months were reported for each study, with the mean IOP difference attributable to each of the devices calculated and the results meta-analysed for all the included studies which reported data at that time point (Figure 4).

Although not statistically significant, the mean baseline IOP was numerically higher in the MINIject compared to the iStent cohort (23.7 vs. 21.1 mmHg respectively; *p* = 0.07), which may have contributed to a greater magnitude of mean absolute IOP reduction. At 24 months, the mean change in baseline IOP in the MINIject cohort was −9.57 mmHg (95% CI: −10.73, −8.41) compared with −4.92 mmHg (95% CI: −8.86, −0.98) in the iStent cohort, which was statistically significant (*p* = 0.03).

There was a statistically greater reduction in IOP from baseline to 24 months for MINIject vs. two iStents of 4.65 mm Hg (*p* = 0.03). The mean percentage IOP reduction at 24 months ranged from 36.3% to 42.2% in the MINIject studies, compared with 5.2% to 40.7% in the iStent studies. A greater variability in the mean change in IOP from baseline was evident in the iStent meta-analyses; the I^2^ measure showed considerable heterogeneity, with I^2^ = 96.53% for iStent at the 24-month follow-up point. In contrast, the MINIject studies showed minimal/no heterogeneity with I^2^ = 0%.

### 3.3. Meta-Analyses of IOP-Lowering Medication Reduction in MINIject vs. iStent up to 24 Months

The mean values for IOP-lowering medication use at baseline and at 24 months were extracted for each study, with the mean medication use difference attributable to each of the devices calculated and the results meta-analysed for all the included studies which reported data at that time point (Figure 5).

**Figure 2:**
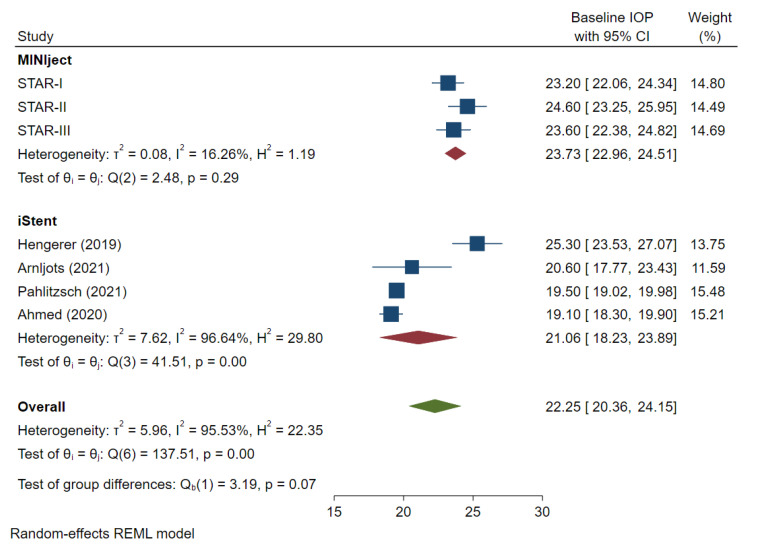
Forest plot evaluating mean baseline intraocular pressure in each study. IOP: intraocular pressure. The mean difference and 95% CI are calculated as a mean difference using *t*-test. “Test of group differences” provides the *p*-value for the indirect treatment comparison analysis [8,19,20,21].

One study which reported IOP outcomes did not report IOP-lowering medication use at the 24-month follow-up point [18,19]. At baseline, there was no difference (*p* = 0.39) in the mean medication use between the MINIject (2.4) and the iStent (2.7) studies (Figure 2). At 24 months, the change from baseline in mean medication use was −1.00 (95% CI −1.67, −0.32) for MINIject and −0.56 (95% CI −0.89, −0.23) for iStent (*p* = 0.26).

There was a mean between treatment difference for medication use of −0.44 in favour of MINIject, such that MINIject reduced medication use by 0.44 medications more than two iStents at 24 months. However, this difference was not statistically significant (*p* = 0.26). Variability was present for both devices, with high variability of I² = 64.80% for MINIject and moderate variability of I² = 35.03% for two iStents [17].

### 3.4. Assessment of Bias and Study Quality

The key risk of bias in the analysis arose from the requisite indirect nature of the treatment comparison (Appendix A). A comprehensive quality assessment of each included study was conducted using Cochrane ROBINS-1 criteria [24]. Overall, most studies were rated as having a low risk of bias, with one MINIject and two iStent studies having some elements indicating a moderate risk of bias (Table 4).

### 3.5. Comparison of Safety

A safety analysis was performed for all identified studies to supplement the reported efficacy result. In a pooled analysis of the STAR-I,II,III trials [23], frequently reported AEs (N ≥ 5%) related to the MINIject device or surgical procedure were mostly transient (<30 days) and resolved without sequelae, including anterior chamber inflammation (n = 20, 24.4%), BCVA loss (n = 14, 17.1%), hyphema (n = 11, 13.4%), and conjunctival hemorrhage (n = 5, 6.1%). In the iStent studies, frequently reported events (n ≥ 5%) included device obstruction (10/77, 13.0%), BCVA loss (4/44, 9.1%), IOP spike (4/77, 5.2%), and cataract progression (6/121, 5.0%).

Endothelial cell density (ECD) loss has been a concern with prior implants in the suprachoroidal space. Two-year central ECD loss after standalone MINIject implantation was reported to be 6.2% [23]. ECD loss after standalone iStent implantation was not reported in any of the iStent trials identified in this study. The rate of ECD loss two years after iStent implantation in combination with cataract surgery from the pivotal iStent study, as reported to the Food and Drug Administration (FDA), was 13.1% [25].

## 4. Discussion

While numerous studies have demonstrated the IOP efficacy of MIGS devices combined with phacoemulsification, the magnitude of IOP lowering with MIGS devices alone remains less clear. Cataract surgery alone can decrease IOP in patients with elevated IOP [16]. In a study of participants in the Ocular Hypertension Treatment study who underwent cataract surgery, the average decrease in post-operative IOP from baseline was 16.5%, and this effect extended for at least 36 months [16]. Studies on standalone MIGS are therefore important to help us better understand the effect of these devices on IOP. Furthermore, while studies often report the IOP efficacy of MIGS devices at 12 months, few studies report longer-term outcomes. In this meta-analysis, we sought to examine the efficacy of MINIject at 24 months and chose the iStent as the comparator because of its global popularity, the volume of outcome data in published studies, and the length of post-operative follow-up available [26].

### 4.1. Comparison of MINIject vs. iStent

In this meta-analysis at 24 months, there was a statistically greater reduction in IOP from baseline in the MINIject cohort compared with the iStent cohort (−9.57 vs. −4.92 mmHg; *p* = 0.03). Notably, the baseline mean IOP in the Miniject was higher than in the iStent cohort, although this was not statistically significant. The range of percentage IOP reduction in the former was narrower than the iStent cohort, which may indicate a more consistent decrease in IOP. At 24 months, the change from baseline in mean medication use was −1.00 with MINIject and −0.56 medications with iStent, but this difference of −0.44 was not statistically significant (*p* = 0.26).

Notably, the inter-study variability of the reduction in mean IOP was greater for iStent than for MINIject. The mean percentage IOP reduction at 24 months ranged from 36.3% to 42.2% in the MINIject studies compared to 5.2% to 40.7% in the iStent studies. This suggests that the MINIject implant may provide a more consistent reduction in IOP. The I² measure of between-study variability supports this conclusion, with MINIject having an I² = 0% compared with an I² = 96.53% for the iStent studies. There was between-study variability in medication reduction for both devices (I² = 64.8% for MINIject and I² = 35.05% for iStent studies). This is likely to reflect different starting medication levels between the studies (mean range 2–3 for MINIject and 2.2–3 for iStent), as well as differing physician treatment practices in assessing target IOP and adjusting medication levels accordingly across geographies and, thus, between studies.

The greater variability in IOP lowering between studies in iStent may be explained physiologically by the location and functionality of collector channels. Up to 90% of collector channels in POAG eyes may be obstructed by meshwork herniations [27], which limits the effectiveness of a trabecular meshwork bypass into Schlemm’s canal if collector channels are obstructed. In addition, the locations of collector channels are difficult to identify peri-operatively, and research suggests that the effectiveness of micro-bypass stenting is correlated with the proximity of collector channels to the implant location [28,29]. If the stent is placed in a region where the canal has collapsed or prolapsed into the ostia of collectors, the stent may be less effective [30,31]. In addition, Schlemm’s canal interventions to lower IOP are limited by episcleral venous pressure (EVP), which creates an IOP floor, so there may be a limit to how much trabecular meshwork bypass devices can achieve in lowering IOP [15]. Studies utilising anterior-segment optical coherence tomography in glaucoma surgery may be used to visualise microstructural changes to better understand aqueous outflow and also verify the location of device placement, which has been employed in iStent surgery [32,33,34].

A safety comparison between the two devices was difficult to perform due to the difference in safety reporting between the various studies and the different definitions of adverse events. Consistent with the expected outcomes for MIGS procedures, the adverse events reported for both devices were primarily transient in nature, and without sequelae. Although the reported events were different between devices, these were not sight-threatening and were typical of MIGS procedures.

### 4.2. Efficacy of iStent

In a study of 203,146 eyes in the American Academy of Ophthalmology (AAO) IRIS^®^ Registry (Intelligent Research in Sight) that underwent surgery between 2013 and 2018, the iStent was the most commonly performed MIGS [10]. The iStent also has the most literature supporting its efficacy among MIGS devices [26]. The use of the iStent inject in combination with phacoemulsification has demonstrated significant and sustained IOP and medication reduction. In a case series of 124 eyes with various glaucoma subtypes, Salimi et.al. reported a reduction in mean IOP from 16.9 mmHg to 13.2 mmHg and reduction in medication use from 2.4 to 1.2 at 3 years, with 96% of eyes achieving an IOP ≤ 18 mmHg [35]. The efficacy of iStent combined with phacoemulsification is supported by various prospective and retrospective studies of varying follow-up durations [11].

Use of the iStent independent of phacoemulsification has also demonstrated efficacy in lowering IOP and medication burden. In a meta-analysis of studies reporting outcomes of standalone iStent devices, a mean IOP reduction of 7.01 mmHg and 31.1% was observed at 6 to 12 months [36]. Studies with longer-term follow-up periods of between 36 to 60 months reported a mean IOP reduction of 6.59 mmHg, and between 30.4% to 32.9% [36]. The latter meta-analysis, however, included both first- and second- generation iStents, such that studies where one to three iStents were implanted were all included in the meta-analyses with their associated patient populations. Our study only included studies with two iStents implanted, which best matched the mild-to-moderate patient population of the MINIject studies. The iStent meta-analysis also combined studies of various follow-up durations into two groups: “short-term” (6–12 months) or “long-term” (36–60 months), whereas this study only considered one follow-up time point of 24 months.

### 4.3. Shunting Aqueous Flow to the Suprachoroidal Space

Suprachoroidal implants reduce IOP by channelling aqueous humour out of the anterior chamber to the supraciliary space, thus enhancing physiological uveoscleral outflow. The idea of increasing aqueous outflow through the suprachoroidal space dates back more than one century [37]. This followed observations of profound drops in IOP following incidental cyclodialysis cleft formation [37]. In 1905, Leopold Heine developed a spatula to intentionally create a cyclodialysis cleft to shunt aqueous out of the anterior chamber [38]. The ciliary body represents the site of greatest resistance to outflow in the uveoscleral pathway, and a barrier to the suprachoroidal space. The latter acts as a sieve as aqueous permeates scleral vessels, the choriocapillaris, or scleral pores to access episcleral tissue [37]. Uveoscleral outflow is mostly driven by differences in hydrostatic pressure between the anterior chamber and the suprachoroidal space [39]. The removal of the barrier to the suprachoroidal space via an implant may therefore enable the effective lowering of elevated IOP driven by the gradient in hydrostatic pressure, without the IOP floor limitation of episcleral venous pressure that exists in the conventional outflow pathway.

Various devices targeting the suprachoroidal space via an ab interno or ab externo approach have since been developed, with varying levels of success [37,40,41]. The CyPass Microstent (Alcon, Vernier-Geneva, Switzerland) and the iStent Supra (Glaukos Corporation, San Clemente, CA, USA) are MIGS devices which are delivered via an ab interno approach into the suprachoroidal space, although neither is commercially available. The CyPass microstent was approved for use in conjunction with cataract surgery in patients with mild-to-moderate primary open-angle glaucoma by the FDA in July 2016. This was based on two-year outcomes of the COMPASS study demonstrating a significant reduction in IOP compared to cataract surgery control [42]. The CyPass micro-stent was, however, voluntarily withdrawn from the global market in 2018 due to statistically significant endothelial cell loss at the 5-year follow-up point compared to the control group [43].

**Figure 3:**
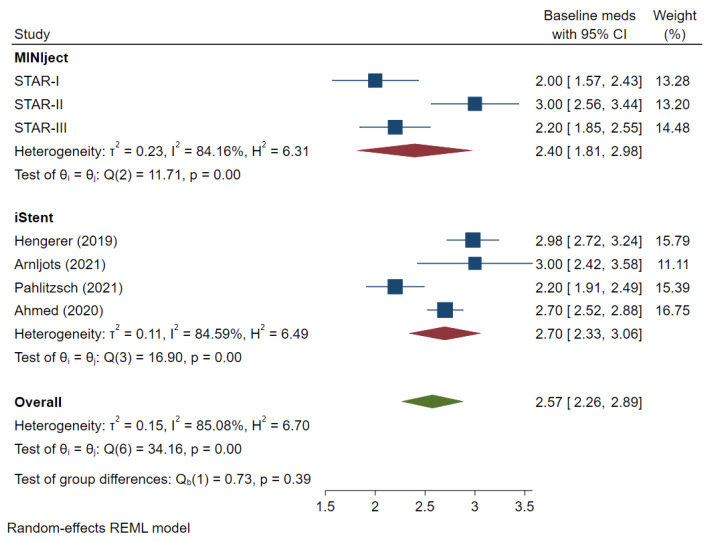
Forest plot evaluating mean baseline ocular glaucoma medications used in each study. IOP: intraocular pressure. The mean difference and 95% CI are calculated as a mean difference using *t*-test. “Test of group differences” provides the *p*-value for the indirect treatment comparison analysis [8,19,20,21].

**Figure 4:**
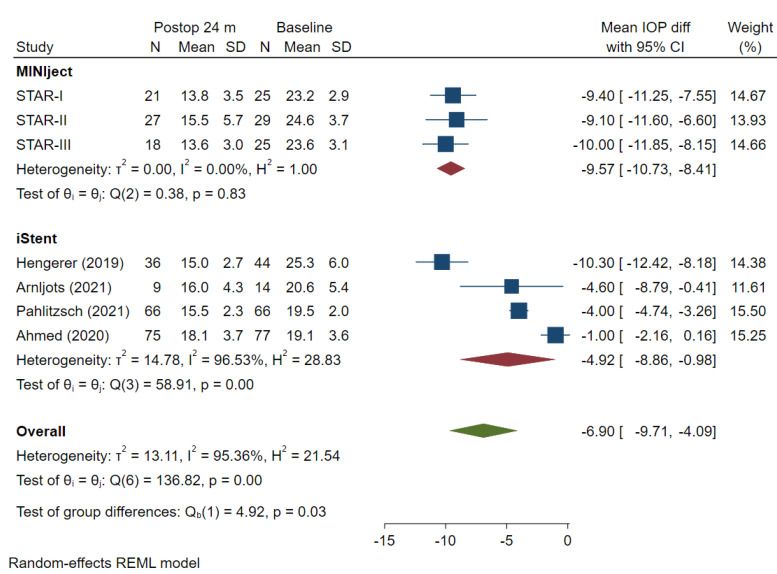
Mean change from baseline in IOP at 24 months (mmHg). IOP: intraocular pressure. The mean difference and 95% CI are calculated as a mean difference using *t*-test. “Test of group differences” provides the *p*-value for the indirect treatment comparison analysis [8,19,20,21].

**Figure 5:**
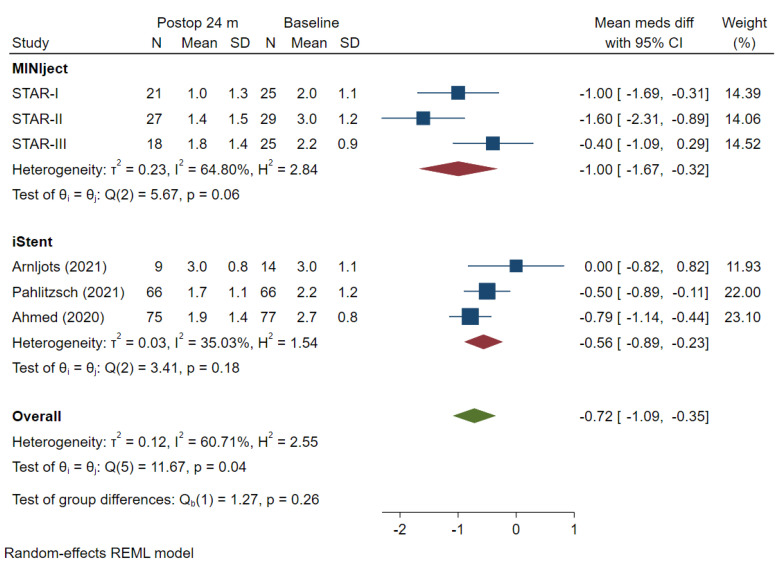
Mean change from baseline to month 24 in number of IOP-lowering medications. IOP: intraocular pressure. The mean difference and 95% CI are calculated as a mean difference using *t*-test. “Test of group differences” provides the *p*-value for the indirect treatment comparison analysis [8,20,21].

A pooled meta-analysis of 24-month results of MINIject in 66 patients from the STAR-I,II,III trials has been published recently [23]. Two-year MINIject outcomes demonstrated a mean 39.3% IOP reduction from baseline IOP of 23.8 ± 3.3 to 14.4 ± 4.5 mmHg (−9.6 mmHg; *p* < 0.0001) and a reduction in medications from 2.4 ± 1.1 to 1.4 ± 1.4 at 24 months. These published results are consistent with the results shown in this meta-analysis, which were obtained from the manufacturer’s database, where a mean IOP reduction of −9.57 mmHg (Figure 3) and a mean reduction in medications of 1.00 (Figure 4) were calculated.

### 4.4. Limitations

While the use of data from the manufacturer’s database could have introduced bias towards MINIject in this analysis, this effect was mitigated through a comparison with the subsequent published pooled data of the same studies, which corroborated the results produced by this analysis that used internal data. Another limitation could exist in that the mean baseline IOP was numerically higher in the MINIject studies compared to the iStent cohort, although not statistically significant, which may have contributed to a greater magnitude of mean absolute IOP reduction. The mean baseline intraocular pressure and the number of IOP-lowering medications were 23.7 mmHg (range 23.2–24.6) and 2.4 (range 2.0–3.0) in the MINIject studies and 21.1 mmHg (range 19.1–25.3) and 2.7 (range 2.2–3.0) in the iStent cohort, respectively (*p* = 0.07 for difference in baseline IOP; *p* = 0.39 for difference in baseline medication use). The percentage reduction in IOP may be a more appropriate metric for comparison between both devices; however, as this outcome was not adequately reported (including standard deviation) by the studies in the literature, a valid comparison and analysis was not possible. The MINIject and iStent studies included in the meta-analyses also had small participant sample sizes and did not directly compare both devices; therefore larger randomised or observational studies are required in order to evaluate the standalone efficacy of these devices with greater confidence. In addition, the diagnostic inclusion criteria were not uniform across the seven studies, with a greater heterogeneity of diagnoses included in the iStent population. To address this limitation, a risk of bias assessment was performed using Cochrane ROBINS-1 for non-randomised studies. All three MINIject and three of four iStent studies had a low risk of bias. Another limitation is that two generations of iStent were used in the meta-analysis: the G1 used in the COMPARE trial and the iStent inject. Despite two G1 iStent devices being used in the COMPARE trial, similar to the iStent inject, the differences in the design of these devices could have contributed to some of the differences in outcome. Lastly, there were other standalone iStent studies in the literature with longer-term follow up, which could not be included as 24-month outcomes were either not reported or were reported with different outcomes not including the mean and standard deviation of IOP [44].

## 5. Conclusions

Standalone implantation of a supraciliary device or two trabecular bypass devices resulted in significant reductions in intraocular pressure (IOP) and medication use after 24 months. Supraciliary implants may however provide a greater and more consistent reduction in IOP.

While both MINIject and iStent resulted in significant reductions in IOP and IOP-medication use, standalone MINIject may provide a greater and more consistent reduction in IOP.

## Figures and Tables

**Table 1:** Key baseline characteristics in MINIject and iStent studies.

Study		N Patients	Mean (SD) Age, (Years)	% Male	Lens Status (Percentage Pseudophakic)	Mean (SD) N Medications	Mean (SD) Medicated Baseline IOP, (mm Hg)	Inclusion Criteria
MINIject								
Denis et.al., 2019 (STAR-I) [13,14]		26	69.4 (11.1)	54%	50.0%	2.0 (1.1)	23.2 (2.9)	OAG
Garcia Feijoo et.al., 2020 (STAR-II) [15]		31	69.5 (10.9)	29%	38.7%	3.0 (1.2)	24.6 (3.7)	POAG
Dick et.al., 2024 (STAR-III) [23]		25	66.5 (6.0)	64%	48.0%	2.2 (0.9)	23.6 (3.1)	OAG
iStent								
Ahmed et.al., 2020 (COMPARE) [8]		77	66.5 (9.5)	42%	37.7%	2.7 (0.8)	19.1 (3.6)	OAG
Hengerer et.al., 2019 [19]		44	71.3 (10.5)	52%	NR	2.98 (0.88)	25.3 (6.0)	POAG (86.4%), PXFG (9.1%), other (4.6%)
Arnljots et.al., 2021 [20]		14	72.0 (12.1)	7%	64.3%	3.0 (1.1)	20.6 (5.4)	Mild-moderate OAG or OHT
Pahlitzsch et.al., 2021 [21]		66	76.0 (8.9)	47%	NR	2.2 (1.2)	19.5 (2.0)	POAG

IOP: intraocular pressure; NR: not reported; N: number; SD: standard deviation; OAG: open-angle glaucoma; POAG: primary open-angle glaucoma; PXFG: pseudoexfoliation glaucoma, OHT: ocular hypertension.

**Table 2:** Efficacy results reported for the included MINIject studies.

Study Name and Citation	N Eyes Implanted at Baseline	Follow-Up (Months)	Mean IOP (±SD) Regardless of Meds, (mmHg)	Percent IOP Reduction	IOP ≤ 18 mmHg (%)	Mean Number of Medications (±SD)
MINIject						
Denis et.al., 2019 (STAR-I) [13,14]	25	Baseline	23.2 ± 2.9	-		2.0 ± 1.1
		6	14.2 ± 4.7	39.1%	83%	0.3 ± 0.7
		12	16.0 ± 4.8	30.8%	71%	0.4 ± 0.9
		24	13.8 ± 3.5	40.7%	20/21 (95%)	1.0 ± 1.3
Garcia Feijoo et.al., 2020 (STAR-II) [15]	29	Baseline	24.6 ± 3.7	-	-	3.0 ± 1.2
		6	14.7 ± 6.0	40.2%	79%	0.9 ± 1.2
		12	15.1 ± 5.3	38.3%	86%	1.3 ± 1.4
		24	15.5 ± 5.7	36.3%	20/27 (74%)	1.4 ± 1.5
Dick et.al., 2024 (STAR-III) [23]	25	Baseline	23.6 ± 3.1	-	-	2.2 ± 0.9
		6	14.4 ± 3.5	39.0%	85%	1.4 ± 1.5
		12	15.6 ± 4.4	33.1%	75%	1.4 ± 1.4
		24	13.6 ± 3.0	42.2%	16/18 (89%)	1.8 ± 1.4

N: number; IOP: intraocular pressure; SD: standard deviation.

**Table 3:** Efficacy results reported for the included iStent studies.

Study Name and Citation	N Patients at Baseline	Follow-Up (Months)	Mean IOP (±SD) Regardless of Meds (mmHg)	Percent IOP Reduction	IOP ≤ 18 mmHg, (%)	Mean (±SD) N Medications
iStent						
Ahmed et.al., 2020 (COMPARE) [8]	77	Baseline	19.1 ± 3.6	-	-	2.7 ± 0.8
		6	17.9 ± 3.8	6.3%	NR	1.48 ± 1.25
		12	18.1 ± 3.7	5.2%	57.3%	1.69 ± 1.37
		24	18.1 ± 3.7	5.2%	NR	1.91 ± 1.36
Hengerer et.al., 2019 [19]	44	Baseline	25.3 ± 6.0	-	-	2.98 ± 0.88
		6	NR	-	NR	NR
		12	15.2 ± 3.1	39.9%	90%	NR
		24	15.0 ± 2.7	40.7%	94.4%	NR
Arnljots et.al., 2021 [20]	14	Baseline	20.6 ± 5.4	-	-	3.0 ± 1.1
		6	18.3 ± 2.3	11.2%	NR	NR
		12	18.4 ± 2.4	10.7%	NR	NR
		24	16.0 ± 4.38	22.3%	NR	3.0 ± 0.75
Pahlitzsch et.al., 2021 [21]	66	Baseline	19.5 ± 2.0	-	-	2.2 ± 1.2
		6	14.3 ± 3.0	26.7%	NR	1.5 ± 1.3
		12	14.5 ± 3.0	25.6%	64.2%	1.3 ± 1.2
		24	15.5 ± 2.3	20.5%	NR	1.7 ± 1.1

NR: not reported; N: number; IOP: intraocular pressure; meds: medications; SD: standard deviation.

**Table 4:** Quality assessment results of included studies by ROBINS-1.

Study Name (Data Source)		Confounding	Participant Selection	Intervention Classification	Deviations from Intended Intervention	Missing Data	Outcome Measurement	Selection of Reported Results	Overall
MINIject									
Denis et.al., 2019 (STAR-I) [13,14]		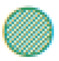	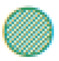	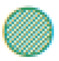	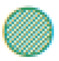	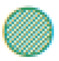	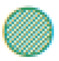	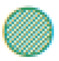	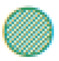
Garcia Feijoo et.al., 2020 (STAR-II) [15]		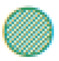	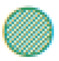	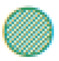	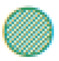	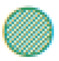	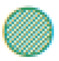	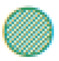	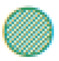
Dick et.al., 2024 (STAR-III)		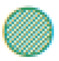	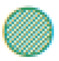	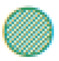	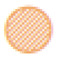	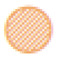	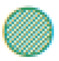	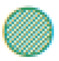	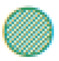
iStent									
Ahmed et.al., 2020 (COMPARE) [8]		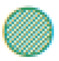	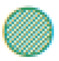	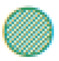	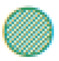	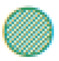	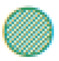	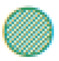	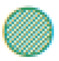
Hengerer et.al., 2019 [19]		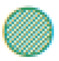	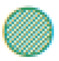	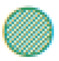	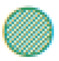	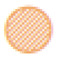	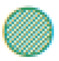	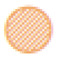	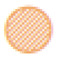
Arnljots et.al., 2021 [20]		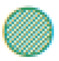	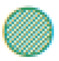	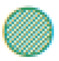	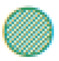	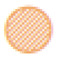	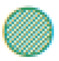	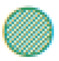	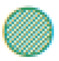
Pahlitzsch et.al., 2021 [21]		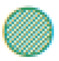	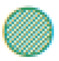	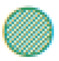	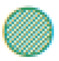	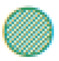	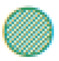	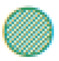	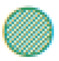

Key: 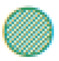 low risk of bias; 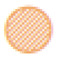 moderate risk of bias. Source: ROBINS-1: Risk of bias in non-randomised studies—of intervention (22).

## Abbreviations

Minimally Invasive Glaucoma Surgery (MIGS), intraocular pressure (IOP).

## Appendix A. Assessment of Study Quality and Bias

Study quality assessments were conducted based on the 24-month key outcome “mean IOP change from baseline”, which was used as the main comparative efficacy outcome in the meta-analysis. There was a low risk of bias from baseline confounding, participant selection, and intervention classification for all the studies, with these being pre-defined criteria that were adequately reported in all studies. A low to moderate risk of bias arose because of deviations from intended interventions, missing data, and selection of reported results. One iStent study did not report IOP-lowering medication use at 24 months, and therefore could not provide outcome data for the medication reduction outcome [18,19], resulting in an overall moderate risk-of-bias rating for that study. Another source of potential bias was the data provided by the manufacturer for unpublished studies -month results of the STAR-II and STAR-III studies). This potential bias was mitigated by accessing the published 24-month results of the pooled STAR-I,II,III trials and comparing the mean baseline and 24-month outcomes with those provided by the manufacturer for individual trials. As these were a match, the internal data provided were considered as having a low risk of bias. All other studies were rated as having a low overall risk of bias based on a comprehensive analysis across the ROBINS-1 study rating criteria [24].
